# Sustainable CO_2_ Capture: N,S-Codoped Porous Carbons Derived from Petroleum Coke with High Selectivity and Stability

**DOI:** 10.3390/molecules30020426

**Published:** 2025-01-20

**Authors:** Jiawei Shao, Yingyi Wang, Mingyang Che, Ya Liu, Yongfu Jiang, Qiang Xiao, Muslum Demir, Linlin Wang, Xin Hu

**Affiliations:** 1 Key Laboratory of the Ministry of Education for Advanced Catalysis Materials, Zhejiang Normal University, Jinhua 321004, China; shaojw2001@outlook.com (J.S.); wangyingyi0@126.com (Y.W.); 13154843717@163.com (M.C.); sky48@zjnu.cn (Y.L.); yongfujiang@zjnu.cn (Y.J.); 2 Institute of Plant Nutrition, Resources and Environment, Beijing Academy of Agriculture and Forestry Science, Beijing 100097, China; 3 Department of Chemical Engineering, Bogazici University, 34342 Istanbul, Türkiye; demirm@alumni.vcu.edu; 4 TUBITAK Marmara Research Center, Material Institute, 41470 Gebze, Türkiye; 5 Key Laboratory of Urban Rail Transit Intelligent Operation and Maintenance Technology and Equipment of Zhejiang Province, College of Engineering, Zhejiang Normal University, Jinhua 321004, China; wanglinlin@zjnu.cn

**Keywords:** N,S-codoped porous carbons, CO_2_ adsorption, petroleum coke, thiourea

## Abstract

CO_2_ capture from the flue gas is a promising approach to mitigate global warming. However, regulating the carbon-based adsorbent in terms of textural and surface modification is still a challenge. To overcome this issue, the present study depicts the development of cost-effective and high-performance CO_2_ adsorbents derived from petroleum coke, an industrial by-product, using a two-step process involving thiourea modification and KOH activation. A series of N,S-codoped porous carbons was synthesized by varying activation temperatures and KOH quantity. The optimized sample exhibited a high specific surface area of 1088 m^2^/g, a narrow micropore volume of 0.52 cm^3^/g, and considerable heteroatom doping (1.57 at.% nitrogen and 0.19 at.% sulfur). The as-prepared adsorbent achieved a CO_2_ adsorption capacity of 3.69 and 5.08 mmol/g at 1 bar, 25 °C and 0 °C, respectively, along with a CO_2_/N_2_ selectivity of 17. Adsorption kinetics showed 90% of equilibrium uptake was achieved within 5 min, while cyclic studies revealed excellent stability with 97% capacity retention after five cycles. Thermodynamic analysis indicated moderate isosteric heat of adsorption (*Q_st_*) values ranging from 18 to 47 kJ/mol, ensuring both strong adsorption and efficient desorption. These findings highlight the potential of petroleum coke-derived porous carbons for sustainable and efficient CO_2_ capture applications.

## 1. Introduction

The escalating levels of atmospheric carbon dioxide (CO_2_), reaching up to 420 ppm in 2024, primarily driven by fossil fuel consumption and industrial activities, represent one of the most pressing challenges of our time [1]. CO_2_, a major greenhouse gas, is the principal driver of global warming and climate change, necessitating the urgent development of mitigation strategies. Among the various approaches to tackle this issue, carbon capture, utilization, and storage (CCUS) technologies are at the forefront, with adsorption-based capture methods garnering particular attention due to their energy efficiency, scalability, and economic viability. Various solid-based materials have been reported as CO_2_ uptake adsorbents such as zeolites [2], metal-organic frameworks (MOFs) [3,4], silicas [5], 2D materials [6]_,_ porous polymers [7,8,9], and carbons [10,11,12,13,14,15,16].

Among those, porous carbon materials have emerged as highly promising adsorbents for CO_2_ capture, thanks to their unique properties, including high surface area, tunable pore structures, chemical stability, and ease of functionalization [17,18,19]. However, enhancing their CO_2_ uptake capacity under ambient conditions while maintaining cost-effectiveness remains a great challenge [20]. Microporous carbons, particularly those with narrow micropores (<1 nm), have demonstrated exceptional performance in CO_2_ adsorption owing to their size compatibility with CO_2_ molecules. It was reported that narrow micropores enable stronger physisorption interactions by confining CO_2_ molecules within a space that closely matches their kinetic diameter. This confinement effect enhances the adsorptive capacity, especially under low pressures and room temperature, making such materials highly suitable for practical applications [21,22]. In addition to micropore engineering, surface chemistry plays a pivotal role in improving CO_2_ adsorption performance. Incorporating heteroatoms, such as nitrogen (N) and sulfur (S), into carbon frameworks has been shown to significantly enhance CO_2_ uptake capacity [23,24]. Nitrogen functionalities, such as pyridinic and pyrrolic nitrogen, introduce electron-donating active sites that facilitate sorption of CO_2_ [25,26], while sulfur, explicitly its oxidized forms, increases surface polarity, thereby strengthening physisorption interactions [27]. However, designing materials that effectively integrate narrow microporosity with optimized heteroatom doping remains a significant challenge.

Unlike many previous studies that focus on either porosity or heteroatom doping in isolation, our work integrates these factors to achieve synergistic effects. This dual optimization strategy not only enhances CO_2_ adsorption capacity but also provides a deeper understanding of the mechanisms governing CO_2_ capture. Furthermore, the use of petroleum coke as a precursor adds a sustainable dimension to this study, converting an industrial by-product into high-value materials for environmental applications. Petroleum coke (PC) is a waste by-product generated during the upgrading of heavy oil fractions into lighter, more valuable products to meet the rising demand for light oil. This process, which involves the thermal cracking of residual heavy oil fractions, produces light hydrocarbons in the gas stream while leaving behind a solid. Owing to its high carbon content and low levels of volatiles and ash, PC serves as an excellent carbon precursor for producing porous carbons. With appropriate activation techniques, such as chemical or thermal treatments, this industrial waste can be converted into cost-effective, value-added materials [28,29]. Recently, Jang et al. reported that PC-derived porous carbons for CO_2_ capture via direct KOH activation. The optimal adsorbent achieved a CO_2_ uptake of 3.68 mmol/g at 25 °C and 1 bar. It was noted that narrow micropores (<0.8 nm) were critical for CO_2_ adsorption and high CO_2_/N_2_ selectivity [30]. Furthermore, they prepared porous carbons by a process of carbonization and KOH activation, reaching a maximum CO_2_ adsorption capacity of 4.17 mmol/g at 25 °C and 1 bar, due to the high narrow micropore volume. The materials also showed good CO_2_/N_2_ selectivity, fast kinetics, easy regeneration, and stable cyclic performance, making them promising for practical CO_2_ capture [31]. The findings from these studies have significant implications for the development of scalable, cost-effective, and high-performance adsorbents for carbon capture technologies.

It is important to note that the present study is novel and unique in terms of a comprehensive approach to material design, addressing both pore engineering and surface functionalization in a single framework. Previous studies have predominantly focused on textural characteristics of the carbon-based adsorbent and ignored the importance of surface functionality to some extent. Herein, the main purpose of the present work is to thoroughly evaluate the effects of both textural and surface modifications on CO_2_ capture performance.

In this study, we present a novel strategy for synthesizing N,S-codoped porous carbons derived from petroleum coke, an abundant and low-cost carbon precursor. The synthesis involves a two-step process: thiourea modification to introduce nitrogen and sulfur functionalities, followed by KOH activation to create hierarchical porosity. The systematic tuning of activation temperature and KOH/PC mass ratio enabled us to produce a series of sorbents with tailored porosity and surface chemistry. The integration of narrow microporosity and N,S-codoping resulted in remarkable CO_2_ adsorption performance.

## 2. Results and Discussion

### 2.1. Morphological, Physical and Textural Characterization

**Figure 1:**
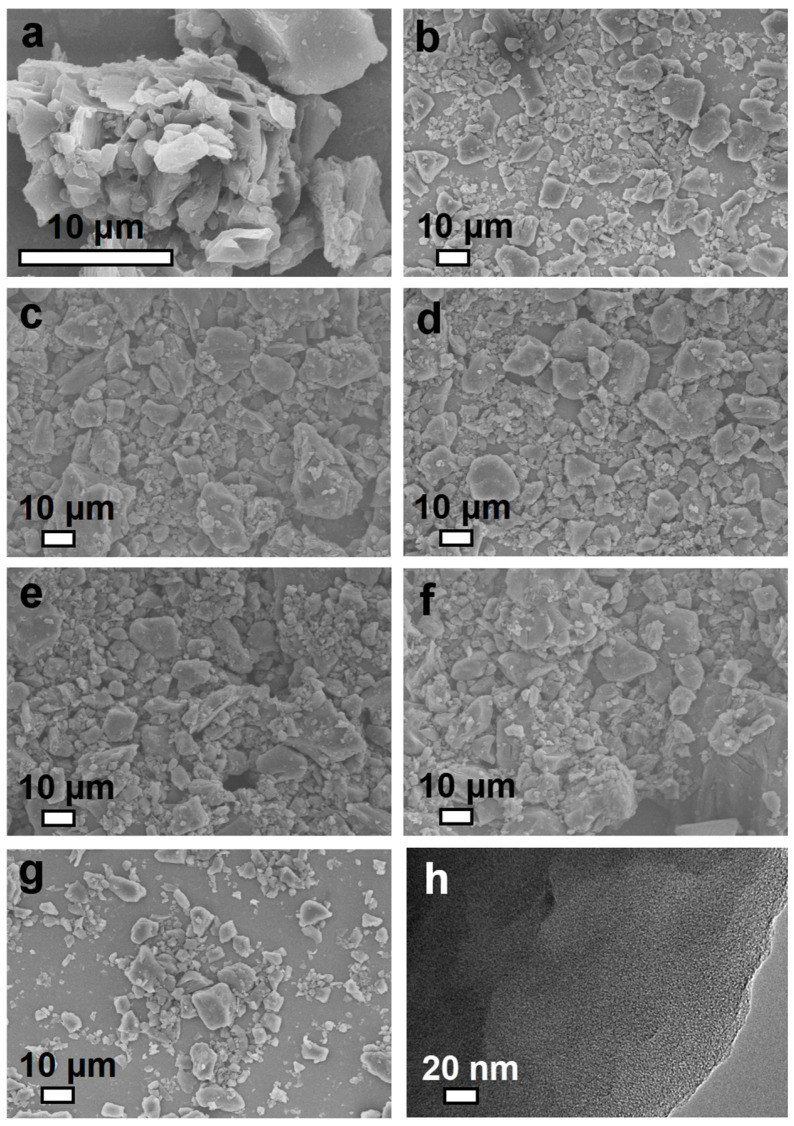
SEM of (**a**) PCT (petroleum coke treated by thiourea), (**b**) PCTK-700-2, (**c**) PCTK-700-3, (**d**) PCTK-700-4, (**e**) PCTK-750-2, (**f**) PCTK-750-3, (**g**) PCTK-750-4, and (**h**) TEM of PCTK-700-4.

The surface morphology and structural characteristics of the samples were investigated using Scanning Electron Microscopy (SEM, Hitachi S-4800, Hitachi, Tokyo, Japan) and Transmission Electron Microscopy (TEM, JEOL-2100F, JEOL, Kyoto, Japan). The SEM image of PC is shown in Figure S1 (Supplementary Materials) revealing a dense and compact morphology, with large and irregularly shaped aggregates, indicative of the untreated nature of the petroleum coke sample. After thiourea modification, the surface morphology of PCT (Figure 1a) did not change much compared to PC. In contrast, the SEM images of PCTK-700-2, PCTK-700-3, and PCTK-700-4 (Figure 1b–d) demonstrated progressive structural changes, with the formation of smaller, more uniform particles as a result of thermal and chemical treatment at 700 °C. It has been noted that the samples treated at 750 °C showed further structural refinement, with significantly reduced particle sizes and highly uniform morphologies. High-resolution TEM of PCTK-700-4 (Figure 1h) revealed a detailed view of its porous structure, characterized by interconnected micropores and an amorphous carbon framework. These observations indicate the successful generation of a porous network that is essential for improving surface area and accessibility, potentially enhancing the material’s performance in CO_2_ uptake activity.

**Figure 2:**
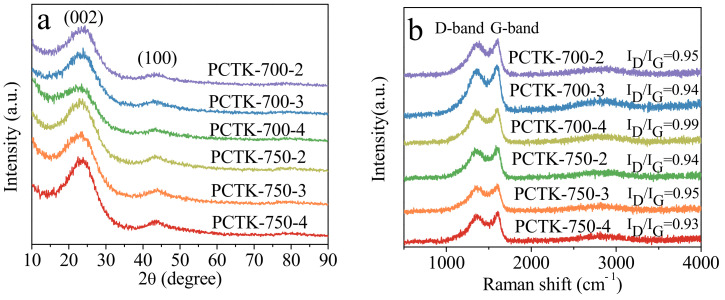
(**a**) XRD patterns and (**b**) Raman spectra of N,S-codoped porous carbons.

The powdered X-ray diffraction (XRD) patterns (Figure 2a) of the N,S-codoped porous carbon samples (PCTK-T-m series) show characteristic peaks at approximately 2θ ≈ 23° and 44°, corresponding to the (002) and (100) planes of graphitic carbon. The broad peak at 23° indicates the presence of turbostratic carbon layers with a highly disordered structure, which is typical for activated carbons. This structural disorder is beneficial for CO_2_ adsorption, as it increases the availability of micropores for gas molecules. For the samples activated at 700 °C (PCTK-700-2, PCTK-700-3, and PCTK-700-4), the XRD patterns reveal relatively broad and low-intensity peaks, confirming a highly amorphous structure. As the activation temperature increases to 750 °C (PCTK-750-2, PCTK-750-3, and PCTK-750-4), a slight sharpening of the (002) peak is observed, suggesting a partial increase in graphitization. This denotes that higher temperatures promote better alignment of carbon layers while still retaining significant disorder. Such a balance between amorphous and graphitic domains is critical, as the amorphous regions contribute to microporosity, and graphitic domains improve thermal stability. Additionally, the effect of the KOH/PCT ratio is evident in the patterns. Samples with higher KOH/PCT ratios, such as PCTK-700-4 and PCTK-750-4, show enhanced structural refinement, likely due to more effective etching and pore development by KOH at higher ratios. Raman spectroscopy provides insights into the structural disorder and graphitization of the samples. The spectra of all PCTK-T-m samples (Figure 2b) exhibit two prominent bands: the D-band (~1350 cm^−1^), associated with defects and disordered carbon, and the G-band (~1580 cm^−1^), corresponding to graphitic sp^2^-hybridized carbon. The relative intensity ratio of the D-band to G-band (I_D_/I_G_) reflects the degree of disorder in the carbon structure. For the PCTK-700-4 sample, the I_D_/I_G_ ratios are close to 1, indicating a high degree of disorder. This is consistent with the broad XRD peaks, which suggest an amorphous structure. The rest of the samples present a similar trend where the I_D_/I_G_ ratios range between 0.93 to 0.95. The above XRD and Raman results highlight that both activation temperature and KOH/PCT ratio influence the crystallinity and pore structure of the carbon materials, which are essential for optimizing CO_2_ adsorption.

**Figure 3:**
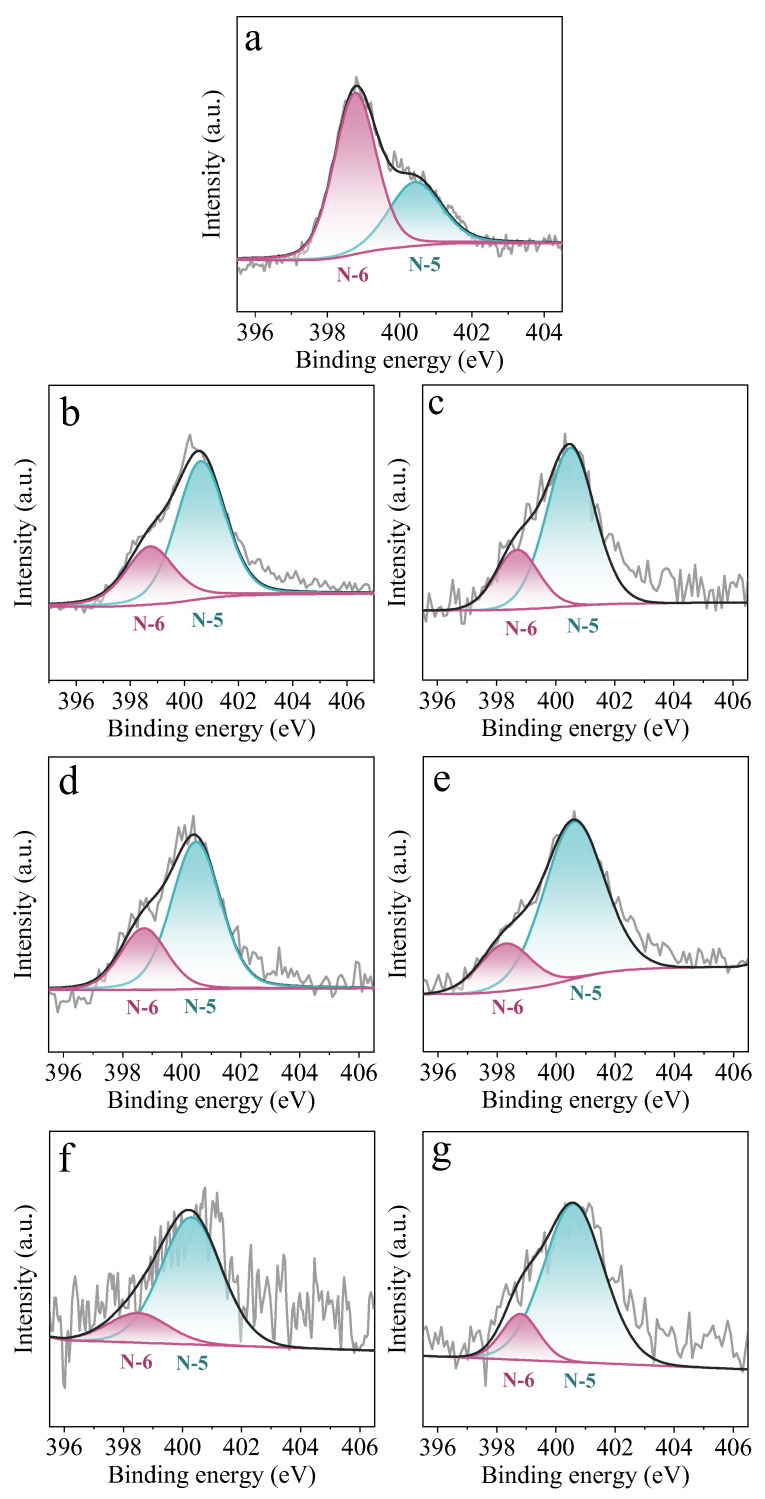
XPS N 1s of (**a**) PCT (petroleum coke treated by thiourea), (**b**) PCTK-700-2, (**c**) PCTK-700-3, (**d**) PCTK-700-4, (**e**) PCTK-750-2, (**f**) PCTK-750-3, and (**g**) PCTK-750-4.

**Figure 4:**
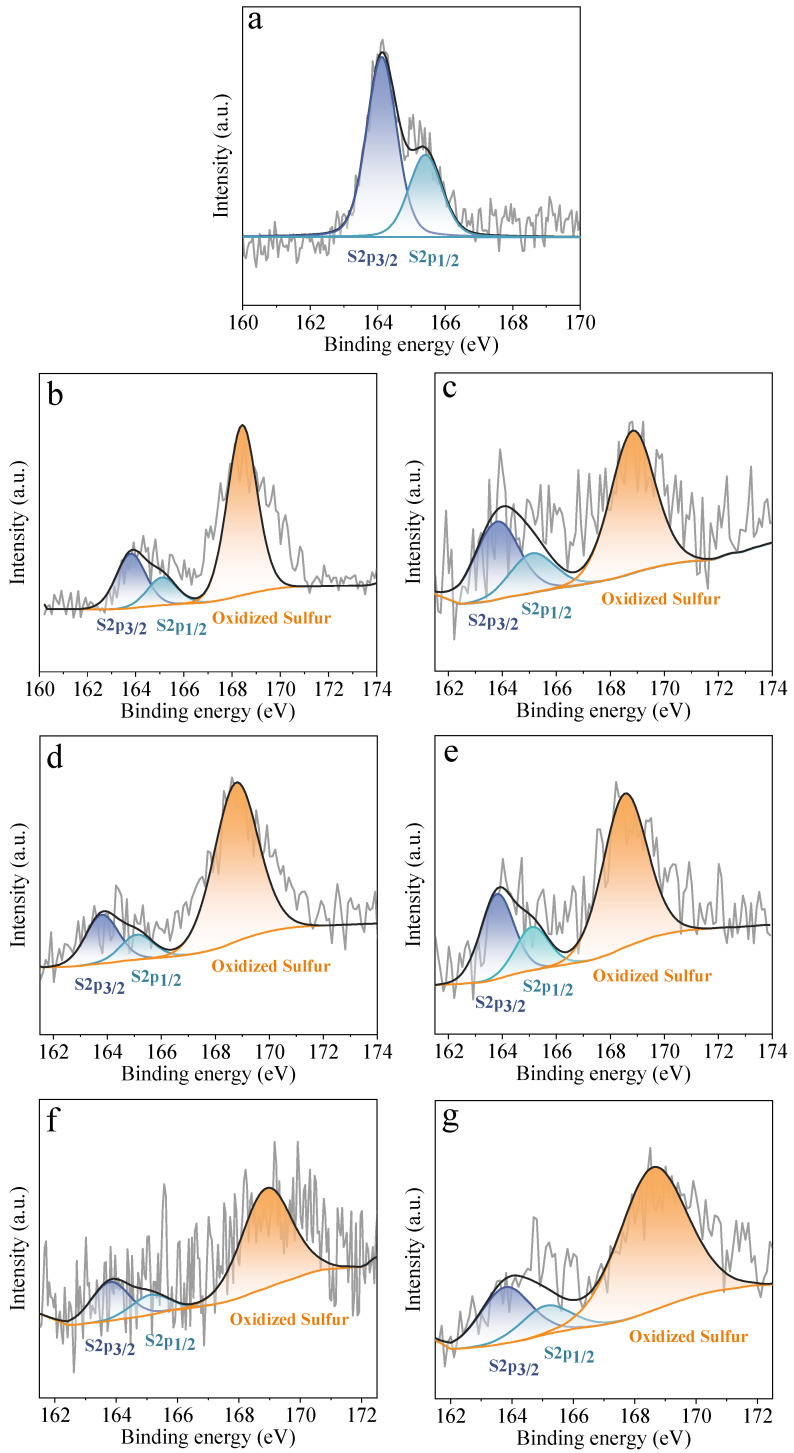
XPS S 2p of (**a**) PCT (petroleum coke treated by thiourea), (**b**) PCTK-700-2, (**c**) PCTK-700-3, (**d**) PCTK-700-4, (**e**) PCTK-750-2, (**f**) PCTK-750-3, and (**g**) PCTK-750-4.

X-ray photoelectron spectroscopy (XPS) was performed to evaluate the elemental composition of PC, PCT, and PCTK-T-m adsorbents. Figure S2 shows the XPS survey scan spectrum of PCT and PCTK-T-m samples, which are mainly composed of C, N,S, and S elements. The carbon (C), sulfur (S), nitrogen (N), and oxygen (O) contents of all these adsorbents are provided in Table 1. The PC is mainly composed of C and O, with a small amount of N and S. PC’s high content of C (85.79 at.%) indicates that it can be directly used as a carbon precursor without the extra carbonization process. After the thiourea treatment, PCT shows a great increase in the N content and a slight increase in the S content, which indicates both N and S have been integrated into the carbon framework by thiourea modification. After KOH activation at elevated temperatures, the N content decreased with the increase of activation temperature and KOH/PCT ratio, while the S content remained relatively stable across all samples. This is most probably caused by the S doping level being much less than that of N doping in PCT. During the high-temperature pyrolysis process, there is not much difference in the decomposition/consumption of S functionalities for each sample. To further clarify the chemical states of the components on the surface of the adsorbents, Figure 3 illustrates the XPS N1s spectra of various samples treated under different conditions, highlighting the nitrogen functionalities present in these materials. The peaks at 398.7 eV (N-5) correspond to pyrrolic nitrogen, while those at 400.5 eV (N-6) represent pyridinic nitrogen, both of which play significant roles in CO_2_ capture. Pyridinic nitrogen (N-6) is particularly critical due to its lone electron pairs, which enhance the basicity of the material and facilitate strong interactions with CO_2_ through electron-donor mechanisms. On the other hand, pyrrolic nitrogen (N-5) modifies the electronic properties of the material, contributing to surface interactions with CO_2_ and complementing the adsorption process. For PCT, N-6 is the major N species, while for all the PCTK-T-m adsorbents, the amount of N-5 is higher than that of N-6. The presence of both pyridinic and pyrrolic nitrogen in these materials underscores their potential for effective CO_2_ capture applications [32,33]. Figure 4 presents the XPS S 2p spectra of various samples treated under different conditions, showcasing the sulfur functionalities in these materials. The peaks at 163.8 eV (S 2p 3/2) and 165.1 eV (S 2p 1/2) are attributed to thiophenic sulfur, whereas the peak at 168.5 eV corresponds to oxidized sulfur species. It can be observed that PCT only contains thiophene sulfur species, whereas the PCTK-T-m adsorbents contain oxidized sulfur as the major S species. It has been noted that oxidized sulfur is known to improve surface polarity and interaction with polar molecules such as CO_2_, enhancing the material’s adsorption capabilities. Notably, the higher oxidized sulfur content in these samples may increase the material’s ability to interact with CO_2_, making them promising for CO_2_ capture. The presence of both thiophenic and oxidized sulfur underscores the multifunctionality of these materials, enabling them to serve as CO_2_ adsorption sites.

### 2.2. Porous Properties

**Figure 5:**
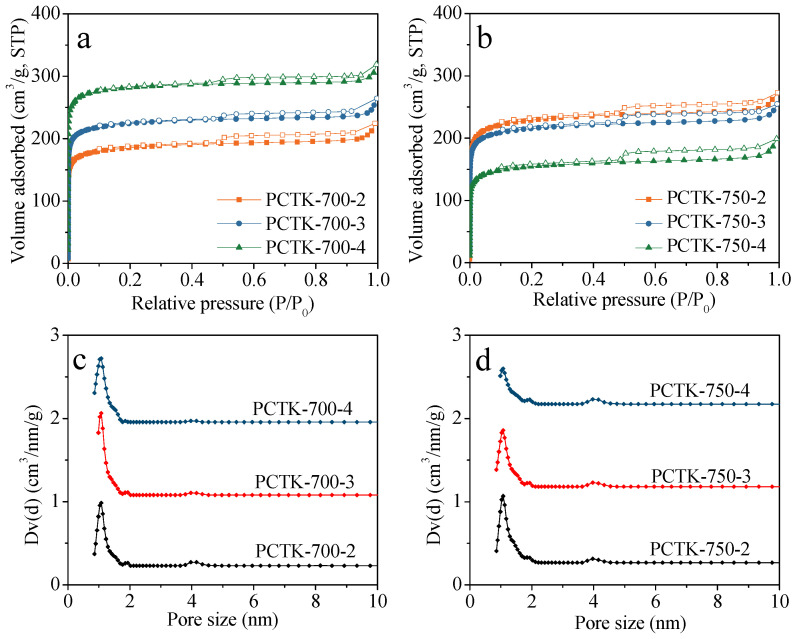
(**a**,**b**) N_2_ sorption isotherms and (**c**,**d**) pore size distribution of the samples prepared at different conditions. Filled and empty symbols in (**a**,**b**) represent adsorption and desorption branches, respectively.

Figure 5 provides a comprehensive analysis of the N_2_ sorption isotherms (a,b) and pore size distributions (c,d) of the samples prepared under various conditions. The adsorption and desorption branches, represented by filled and empty symbols, respectively, exhibit mostly characteristic Type I isotherms, indicative of microporous structures. The slight presence of hysteresis loops in the isotherms confirms the mesoporosity, which is essential for applications requiring high surface area and hierarchical pore structures. The pore size distributions (Figure 5c,d) highlight variations in pore structure across the samples, directly influenced by synthesis conditions such as activation temperature and KOH/PCT ratio. As clearly observed, the dominant micropore size is around 1.1 nm, with humps detected at 4.1 nm corresponding to the mesopore region, which aligns well with the isotherms. The synergy between the high surface area and the well-defined micro/mesoporous structures in these samples underscores their potential for CO_2_ capture. Table 1 presents the porous textural characteristics of these adsorbents i.e., BET surface area (*S*_BET_), total pore volume (*V*_0_), and microporous volume (*V*_t_). PC and PCT possessed negligible porosity, with *S*_BET_ of 11 and 2 m^2^/g, respectively. After KOH activation, the resultant PCTK-T-m samples displayed a well-developed porous structure, with a maximum *S*_BET_ of 1088 m^2^/g and *V*_0_ and *V*_t_ of 0.48 and 0.42 cm^3^/g, respectively. At the activation temperature of 700 °C, the higher KOH/PCT ratio improved the material’s porosity. On the other hand, at the activation temperature of 750 °C, the increase of the KOH/PCT ratio deteriorated the porosity of the adsorbents. The drop in porosity could be attributed to the increase in the KOH/PCT ratio at higher activation temperatures, which led to the collapse of some pore walls during KOH activation [34]. From earlier studies, narrow micropore (*V*_n_) (< 1nm) rather than *S*_BET_, *V*_0,_ and *V*_t_, is the chief factor that decides the CO_2_ uptake of carbonaceous materials under 1 bar and 25 °C [21,22]. Therefore, the *V*_n_ of these PCTK-T-m samples was calculated using the Dubinin−Radushkevich (D−R) equation based on their CO_2_ adsorption data at 0 °C. As shown in Table 1, the *V*_n_ for these samples ranges from 0.40 to 0.52 cm^3^/g.

### 2.3. CO_2_ Adsorption Performance

**Figure 6:**
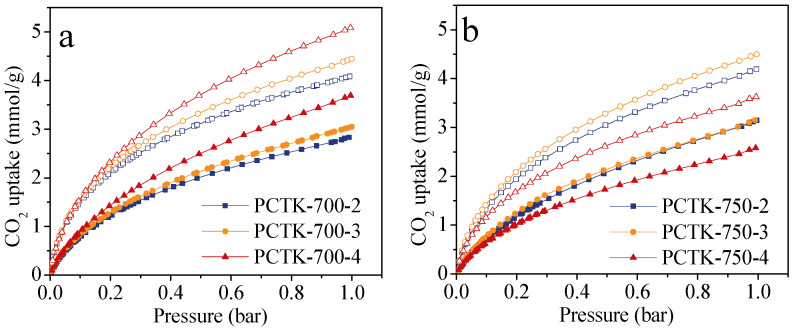
CO_2_ adsorption isotherms at 25 °C (filled symbols) and 0 °C (empty symbols) for N,S-codoped porous carbons prepared at (**a**) 700 °C and (**b**) 750 °C.

The CO_2_ adsorption isotherms in Figure 6 highlight the influence of activation conditions on the adsorption capacity of the porous carbon materials. The CO_2_ capture values for each sample are provided in Table 1. As is observed, the sample PCTK-700-4 demonstrated the highest CO_2_ uptake of 3.69 and 5.08 mmol/g at 25 °C and 0 °C, 1 bar, outperforming the other adsorbents. This exceptional performance is primarily attributed to its maximum porosity of all the samples, which provides abundant surface sites for CO_2_ adsorption. Additionally, its maximum narrow micropore volume of 0.52 cm^3^/g plays a critical role, as narrow micropores (<1 nm) closely match the size of CO_2_ molecules, enhancing physisorption. Furthermore, the presence of heteroatoms also contributed to the adsorption capacity. The nitrogen content of 1.57 at.%, particularly pyridinic and pyrrolic nitrogen species, provided active sorption sites for stronger interactions with CO_2_. The sulfur content of 0.19 at.% further increased surface polarity and enhanced physisorption interactions. The synergistic combination of an advanced porous structure, optimized narrow microporosity, and N,S-codoping resulted in superior CO_2_ adsorption performance. These findings underscore the importance of carefully balancing physical and chemical properties during material synthesis to achieve high-performance CO_2_ adsorbents. It is critical to note that the CO_2_ uptake capacity of optimal PCTK-700-4 adsorbent is comparable to, or exceeding many typical porous adsorbents such as porous carbons [35,36,37], covalent organic frameworks (COFs) [38], porous polymers [7] and MOFs [3], etc. A detailed comparison between the adsorbents in this study and a variety of other sorbents can be found in Table S1 (Supplementary Materials).

We further investigated the relationship between CO_2_ uptake and various porous characteristics and N,S content of the materials, shedding light on the factors influencing their CO_2_ adsorption performance. First of all, as depicted in Figure S3a–c, CO_2_ uptake shows a moderate positive correlation with *S*_BET_, *V*_0_, and *V*_t_, suggesting that an increase in porosity generally enhances adsorption capacity. However, all the above relationships are not strictly linear, indicating that other parameters, such as narrow microporosity and surface chemistry, also play significant roles in determining the overall CO_2_ capture efficiency. As shown in Figure S3d, narrow micropore volume (*V*_n_) showed a significantly stronger correlation with CO_2_ uptake compared to other porous characteristics. This underscores the critical role of narrow micropores (<1 nm) in CO_2_ capture, as their dimensions are closely aligned with the kinetic diameter of CO_2_ molecules, facilitating stronger physisorption interactions. These findings suggest that designing materials with a higher proportion of narrow micropores could significantly enhance their CO_2_ adsorption capacity, particularly under practical operating conditions. Last but not least, the surface chemistry of porous carbons was further assessed in terms of CO_2_ uptake performance. In Figure S3e, the correlation between nitrogen content and CO_2_ uptake is less definitive, of which an upward trend can be observed firstly, but an excess amount of N content results in a reduction in CO_2_ capture activity. This can be explained by the fact that not all the nitrogen species contribute equally to CO_2_ adsorption. In contrast, Figure S3f demonstrates a clearer positive correlation between sulfur content and CO_2_ uptake. This suggests that sulfur functionalities, particularly oxidized sulfur species such as sulfoxides or sulfates, play a significant role in enhancing adsorption performance. In summary, while both nitrogen and sulfur are important contributors to CO_2_ adsorption, sulfur’s impact appears to be more consistent and uniform, likely due to its ability to modify surface properties beneficial for adsorption. Nitrogen, on the other hand, provides CO_2_ binding sites, but its effectiveness is heavily influenced by the specific type and distribution of nitrogen species present in the material. This highlights the need for precise control of both nitrogen and sulfur functionalities during material synthesis to maximize CO_2_ capture performance. To further highlight the impact of the N,S doping on the CO_2_ adsorption capacity, the CO_2_ uptake of reference sample PCK-700-4 was compared with PCTK-700-4. As recorded in Table 1, the CO_2_ uptake of PCK-700-4 is 3.08 mmol g^−1^ at 25 °C and 1 bar, which is 17% lower than that of PCTK-700-4, respectively (Figure S4). The reasons behind the decrease in CO_2_ uptake come from not only the inferior porosity of PCK-700-4, with N_2_ sorption isotherms of both samples shown in Figure S5, but also the less N,S content of PCK-700-4 than PCTK-700-4.

**Figure 7:**
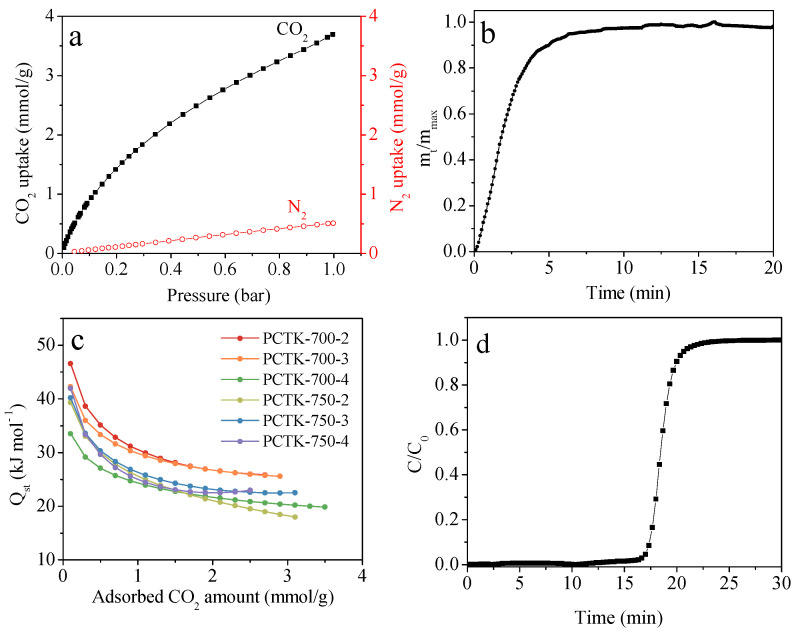
(**a**) CO_2_ and N_2_ isotherms of PCTK-700-4 at 25 °C and 1 bar, (**b**) adsorption kinetic of CO_2_ at 25 °C for PCTK-700-4, (**c**) *Q_st_* of CO_2_ adsorption on PCTK-T-m adsorbents derived from the experimental adsorption isotherms at 0 and 25 °C and (**d**) breakthrough plots of PCTK-700-4 (adsorption temperature: 25 °C, gas flow rate: 10 mL/min, inlet CO_2_ concentration: 10 vol.%, gas pressure: 1 bar).

Figure 7 provides a detailed assessment of the other important CO_2_ adsorption features of the optimal PCTK-700-4 sample, encompassing its CO_2_/N_2_ selectivity, adsorption kinetics, heat of adsorption, and dynamic CO_2_ capture capacity. The CO_2_ and N_2_ adsorption isotherms (Figure 7a) highlight the material’s strong affinity for CO_2_, with a steep initial increase in the isotherm reflecting the presence of narrow micropores and nitrogen/sulfur functionalities, which enhance CO_2_ adsorption. In contrast, N_2_ uptake is significantly lower under identical conditions, resulting in an ideal adsorption solution theory (IAST) CO_2_/N_2_ selectivity of 17. This high selectivity underscores the material’s suitability for separating CO_2_ from N_2_ in flue gases. The adsorption kinetics (Figure 7b) demonstrates the rapid adsorption behavior of PCTK-700-4, with 90% of the equilibrium CO_2_ uptake achieved within approximately 5 min. This fast kinetics can be attributed to the accessible mesoporous structure and high surface area of the material, facilitating efficient CO_2_ diffusion and adsorption. The isosteric heat of adsorption (*Q_st_*) reflecting the strength of interaction between CO_2_ molecules and the adsorbent surface was calculated using the Clausius–Clapeyron equation based on the CO_2_ adsorption isotherms at 25 °C and 0 °C. As shown in Figure 7c, *Q_st_* values range from 34–47 kJ/mol at nearly zero CO_2_ loading for all the samples, indicating relatively strong interaction strength suitable for effective CO_2_ adsorption. However, these values are sufficiently high to ensure effective CO_2_ adsorption but not so strong as to hinder desorption, making the material well-suited for cyclic adsorption-desorption processes. The overall *Q_st_* values, ranging from 18 to 47 kJ/mol, further highlight the balance between strong adsorption capacity and regenerability, making the material practical for cyclic CO_2_ capture applications. The high *Q_st_* at low loading is likely due to the strong interaction between CO_2_ molecules and the nitrogen/sulfur functionalities, which provide strong binding sites. As the adsorption progresses and more sites are occupied, the *Q_st_* decreases. The gradual decrease in *Q_st_* suggests that after the high-energy sites are occupied, CO_2_ molecules interact with less active sites or adsorb within the pores through weaker physical forces. This balance between high initial *Q_st_* and moderate overall *Q_st_* is crucial for practical applications, ensuring sufficient adsorption capacity and efficient regenerability [39,40,41]. The breakthrough curve (Figure 7d) provides insights into the dynamic CO_2_ capture performance of PCTK-700-4. Under a gas flow rate of 10 mL/min, an inlet CO_2_ concentration of 10 vol.%, and a pressure of 1 bar, the material achieves a dynamic capture capacity of 0.93 mmol/g. This value, combined with the rapid breakthrough response, confirms the material’s effectiveness for real-time CO_2_ capture under practical conditions. Collectively, these results demonstrate that PCTK-700-4 is a highly efficient adsorbent for CO_2_ capture, combining high uptake capacity, rapid kinetics, excellent selectivity, promising dynamic capture capacity, and suitable thermodynamic performance.

**Figure 8:**
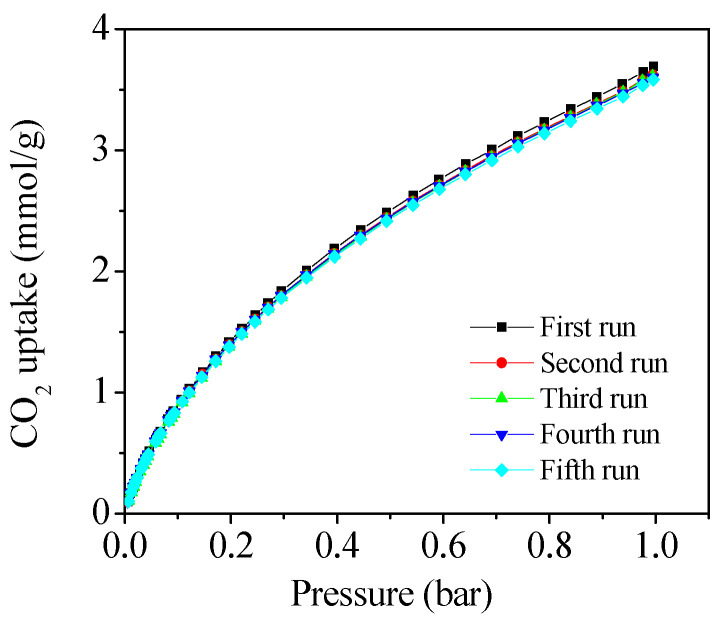
Cyclic study of CO_2_ adsorption for PCTK-700-4.

For practical CO_2_ capture applications, the adsorbent must exhibit both high adsorption capacity and excellent cycling activity and reproducibility. Figure 8 illustrates the cyclic CO_2_ adsorption performance of PCTK-700-4 over five consecutive adsorption–desorption cycles. After five cycles, the sample retains 97% of its original CO_2_ adsorption capacity, indicating minimal performance loss during repeated use. This outstanding cyclic performance is attributed to the robust porous structure and the stability of the nitrogen and sulfur functional groups present in the material.

## 3. Synthesis and Characterization

Petroleum coke (PC) with a particle size of 74–149 μm was used as a carbon precursor to synthesize N,S-codoped porous carbon. First, the same quantity of PC and thiourea was mixed and then heated at 500 °C for 2 h under the N_2_ stream. The resultant N,S-enriched product was denoted as PCT. Next, KOH activation was performed to create porosity of carbon adsorbents. Two temperatures of 700 or 750 °C and three KOH/PCT mass ratios of 2, 3, or 4 were selected during this process. The as-prepared sorbents were designated as PCTK-T-m, of which T and m imply activation temperature and KOH/PCT ratio, respectively. The yield of these N,S-codoped porous carbons ranged from 60.0 to 79.5%. For comparison purposes, a reference sample PCK-700-4 was synthesized as the same procedure as PCTK-700-4 without thiourea modification. The details for material preparation, physical characterization, and CO_2_ adsorption analysis can be found in the Supplementary Materials Section.

## 4. Conclusions

This study demonstrates the successful development of N,S-codoped porous carbons derived from petroleum coke as efficient and cost-effective adsorbents for CO_2_ capture. The optimized sample, PCTK-700-4, exhibited outstanding CO_2_ adsorption performance, attributed to its advanced porosity, narrow micropore volume, and strategically incorporated nitrogen and sulfur functionalities. The integration of these physical and chemical features enabled the material to achieve high adsorption capacity, rapid kinetics, excellent CO_2_/N_2_ selectivity, promising dynamic CO_2_ capture capacity, and stable cyclic performance, making it a strong candidate for practical applications. Additionally, the moderate *Q_st_* values ensured efficient adsorption and desorption processes, further enhancing the material’s feasibility for industrial use. This work highlights the importance of synergistically optimizing textural properties and surface chemistry to maximize CO_2_ capture efficiency. By utilizing petroleum coke, an industrial by-product, as a precursor, this study also contributes to sustainable material design by converting waste into valuable functional materials. The findings provide a robust framework for the development of advanced adsorbents, paving the way for scalable and sustainable solutions to mitigate CO_2_ emissions and combat climate change.

## Figures and Tables

**Table 1:** Porous properties, elemental compositions, and CO_2_ uptakes of N,S-Codoped Porous Carbons.

Sample	S_BET_^a^ (m^2^/g)	V_0_^b^ (cm^3^/g)	V_t_^c^ (cm^3^/g)	V_n_^d^ (cm^3^/g)	XPS (at. %)				CO_2_ Uptake (mmol/g)	
					C	N	S	O	25 °C	0 °C
PC	11	0.01	0	0.16	85.79	1.97	0.24	12.00	0.58	0.85
PCT	2	0.02	0	0.18	79.30	7.94	0.52	12.24	0.80	1.11
PCTK-700-2	699	0.34	0.27	0.40	85.76	3.20	0.17	10.87	2.83	4.09
PCTK-700-3	857	0.40	0.33	0.44	84.05	1.74	0.19	14.02	3.05	4.45
PCTK-700-4	1088	0.48	0.42	0.52	80.37	1.57	0.19	17.88	3.69	5.08
PCTK-750-2	844	0.42	0.33	0.45	88.15	1.93	0.13	9.78	3.14	4.19
PCTK-750-3	807	0.39	0.31	0.46	85.29	1.34	0.19	13.18	3.15	4.50
PCTK-750-4	559	0.30	0.22	0.41	85.94	0.86	0.12	13.08	2.58	3.63
PCK-700-4	787	0.34	0.31	0.49	86.86	1.07	0.18	11.89	3.08	4.54

^a^ Surface area was calculated using the BET method at P/P_0_ = 0.001–0.01. ^b^ Total pore volume at P/P_0_ = 0.99. ^c^ Evaluated by the t-plot method. ^d^ Pore volume of narrow micropores (<1 nm) obtained from the CO_2_ adsorption data at 0 °C.

## Data Availability

The data presented in this study are available on request from the corresponding authors.

## Supplementary Materials

The following supporting information can be downloaded at: https://www.mdpi.com/article/10.3390/molecules30020426/s1, Scheme S1. Schematic diagram of the fixed-bed reactor system, Figure S1: SEM image of PC, Figure S2: XPS survey scan spectrum of PCT and PCTK-T-m samples, Figure S3: Plot of each porous properties characteristics (a) *S*_BET_, (b) *V*_0_, (c) *V*_t_, (d) *V*_n_, (e) nitrogen, and (f) sulfur content versus CO_2_ uptake, Figure S4: CO_2_ adsorption isotherms of PCTK-700-4 vs. PCK-700-4 at 25 °C and 1 bar, Figure S5: N_2_ sorption isotherms of PCTK-700-4 vs. PCK-700-4, Table S1: Comparison of the CO_2_ adsorption (25 °C and 1 bar) for different sorbents. References [42,43,44,45,46,47,48,49,50,51,52,53,54,55] are cited in the Supplementary Materials.
