# Supplementary material for: Sustainable CO2 Capture: N,S-Codoped Porous Carbons Derived from Petroleum Coke with High Selectivity and Stability
Source: Molecules. 2025 Jan 20;30(2):426. doi: 10.3390/molecules30020426 (PMC11767474; doi:10.3390/molecules30020426)
Supplement: Supplementary file 1 [file molecules-30-00426-s001.zip › molecules-3440163-supplementary.pdf]

Sustainable CO_2 Capture: N, S-Codoped Porous Carbons Derived from Petroleum Coke with High Selectivity and Stability

(Supplementary materials)

Jiawei Shao[1], Yingyi Wang[1], Mingyang Che[1], Ya Liu[1], Yongfu Jiang[1], Qiang Xiao[2, *], Muslum Demir[3,4], Linlin Wang[5], Xin Hu[1, *]

1 Key Laboratory of the Ministry of Education for Advanced Catalysis Materials, Zhejiang Normal University, Jinhua 321004, China; shaojw2001@outlook.com (J.S.); wangyingyi0@126.com (Y.W.); 13154843717@163.com (M.C.); sky48@zjnu.cn (Y.L.); yongfujiang@zjnu.cn (Y.J.)
2 Institute of Plant Nutrition, Resources and Environment, Beijing Academy of Agriculture and Forestry Science, Beijing 100097, China
3 Department of Chemical Engineering, Bogazici University, 34342 Istanbul, Türkiye; demirm@alumni.vcu.edu
4 TUBITAK Marmara Research Center, Material Institute, 41470 Gebze, Türkiye
5 Key Laboratory of Urban Rail Transit Intelligent Operation and Maintenance Technology and Equipment of Zhejiang Province, College of Engineering, Zhejiang Normal University, Jinhua 321004, China; wanglinlin@zjnu.cn
* Correspondence: xqiang1978@163.com (Q.X.); huxin@zjnu.cn (X.H.); Tel.: +86-151-0579-0257 (X.H.); Fax: +86-579-8228-8269 (X.H.)

N, S co-doping

A mixture of PC and thiourea (mass ratio 1:1) was heated in N_2 at 500 °C for 2 h. The collected samples were rinsed with hot distilled water a few times to get rid of any unreacted thiourea. Finally, the resulting products were put into the oven to dry at 120 °C overnight. The resulting sample was denoted as PCT.

KOH activation

For a typical reaction, 2 g PCT was combined with a solution that contained 8g KOH. After stirring vigorously for 6 h, the mixture was left overnight to dry at 120 °C in an oven. Afterwards, the sample was activated to 700 °C for 2 h. During the activation process, the heating rate is 5 °C/min and nitrogen flow rate is 400 mL/min. Following activation, the sorbent was rinsed with distilled water until the pH value of the filtrate was roughly 7. The wet sample was then dried at 150 °C under vacuum for 24 h. The obtained sample was denoted as PCTK-700-4.

Characterization

Powdered X-ray diffraction (XRD) patterns were carried out on a PHILIPS PW3040/60 powder diffractometer using CuKα radiation (λ =0.15406nm). Scanning electron microscopy (SEM Hitachi S-4800) was used to observe the morphology of the samples of carbon materials. Further details of the pore structure were determined by transmission electron microscopy (TEM, JEOL-2100F) operated at 200 kV. The CHN elements were analyzed using a VarioEL III Elemental Analyzer. Nitrogen adsorption and desorption isotherms were measured on a Beishide 3H-2000PS2 sorption analyzer at -196°C. Ultrahigh-purity N_2 (99.999%, Shanghai Pujiang Gas Co., Ltd) was used for measurement. Before measurement, the samples were degassed in a vacuum at 200°C for at least 12h. The specific surface area (S_{BET}) was calculated according to the multipoint Brunauer-Emmett-Teller (BET) method from the adsorption data in the relative pressure range between 0.001 and 0.01. The total micropore volume (V_t) was deduced from the N_2 adsorption data by the t-plot method, and the total pore volume (V_0) was estimated from the adsorbed amount of liquid nitrogen at a relative pressure of 0.99. The error of porosity measurement is within 3%. The pore size distribution was calculated using the density functional theory (DFT) method. In addition, X-ray photoelectron (XPS) measurements were performed using an AXIS Nova spectrometer (Kratos Inc., NY, USA) equipped with a monochromatic Al Kα X-ray source (1486.6 eV). XPS survey spectra were recorded with a pass energy of 160 eV, and high-resolution spectra with a pass energy of 40 eV.

The CO_2 adsorption isotherms were measured using the Beshide 3H-2000PS2 sorption analyzer at 0°C and 25°C, respectively. Pure CO_2 (99.99%, Shanghai Pujiang

Gas Co., Ltd) was used for adsorption. Prior to each adsorption experiment, the sample was degassed for 12 h at 200ºC to remove the guest molecules from the pores. The volume of narrow micropores (with sizes <1 nm), V_n, was calculated from CO_2 adsorption at 0°C using the Dubinin–Radushkevich (D-R) equation. The measurements were repeated for each sample, until the values fell within ± 2% of each other.

Measurement of dynamic CO_2 uptake of the sorbents

The dynamic CO_2 uptake of the sorbents was tested on a fixed-bed reactor schematically illustrated in Scheme S1 at 1 bar and 25 °C. First, the sample was heated at 100°C for 1 h under N_2 at a flow rate of 20 mL/min. The gas flow was shifted from nitrogen to a 10% mixture of CO_2 in N_2 at a flow rate of 10 mL/min, when the sample temperature was lowered to 25°C. The effluent gases were monitored online using an Agilent 7820A gas chromatograph with a thermal conductivity detector (TCD). From the breakthrough curves, the dynamic CO_2 capture capacity on an adsorbent was calculated. The error for this measurement falls within 2%.

Measurement of CO_2 adsorption kinetics

The adsorption kinetics of CO_2 was measured in a thermogravimetric analyzer (NETZSCH STA 449C). In the kinetic analysis, the sample (~5 mg) was degassed under a He stream at 200°C for 1 h. Next, the temperature was cooled to the experimental temperature of 25°C. Then the CO_2 gas was fed into the test chamber with a flow rate of 50 mL/min and the weight variation with time was recorded. The measurement' s error is within 2%.

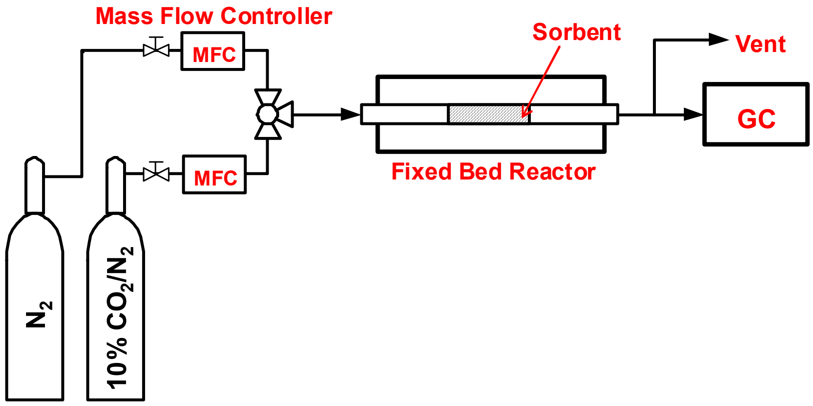

Scheme S1. Schematic diagram of the fixed-bed reactor system.

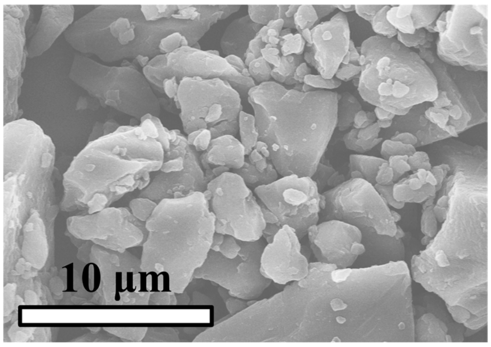

Figure S1. SEM image of PC

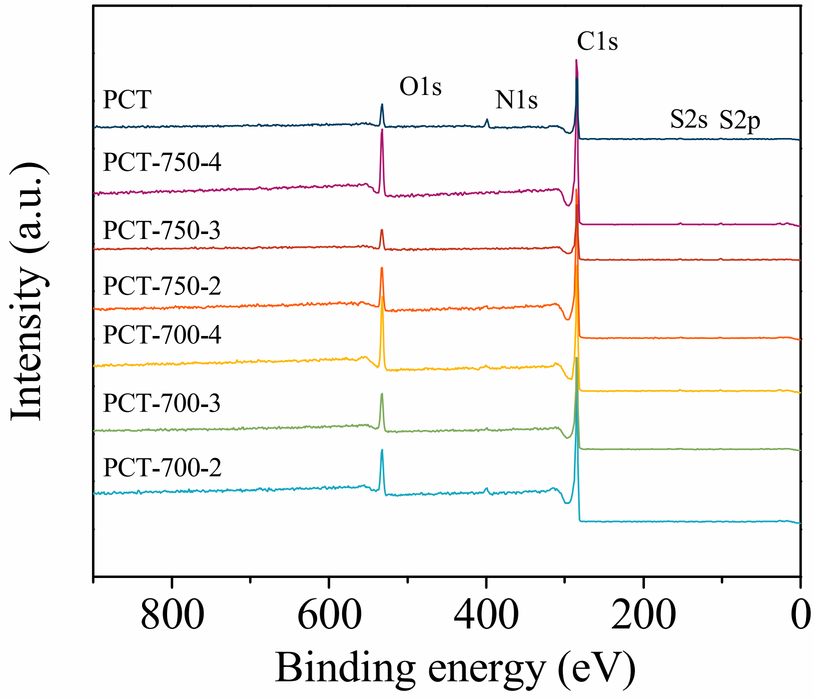

Figure S2. XPS survey scan spectrum of PCT and PCTK-T-m samples

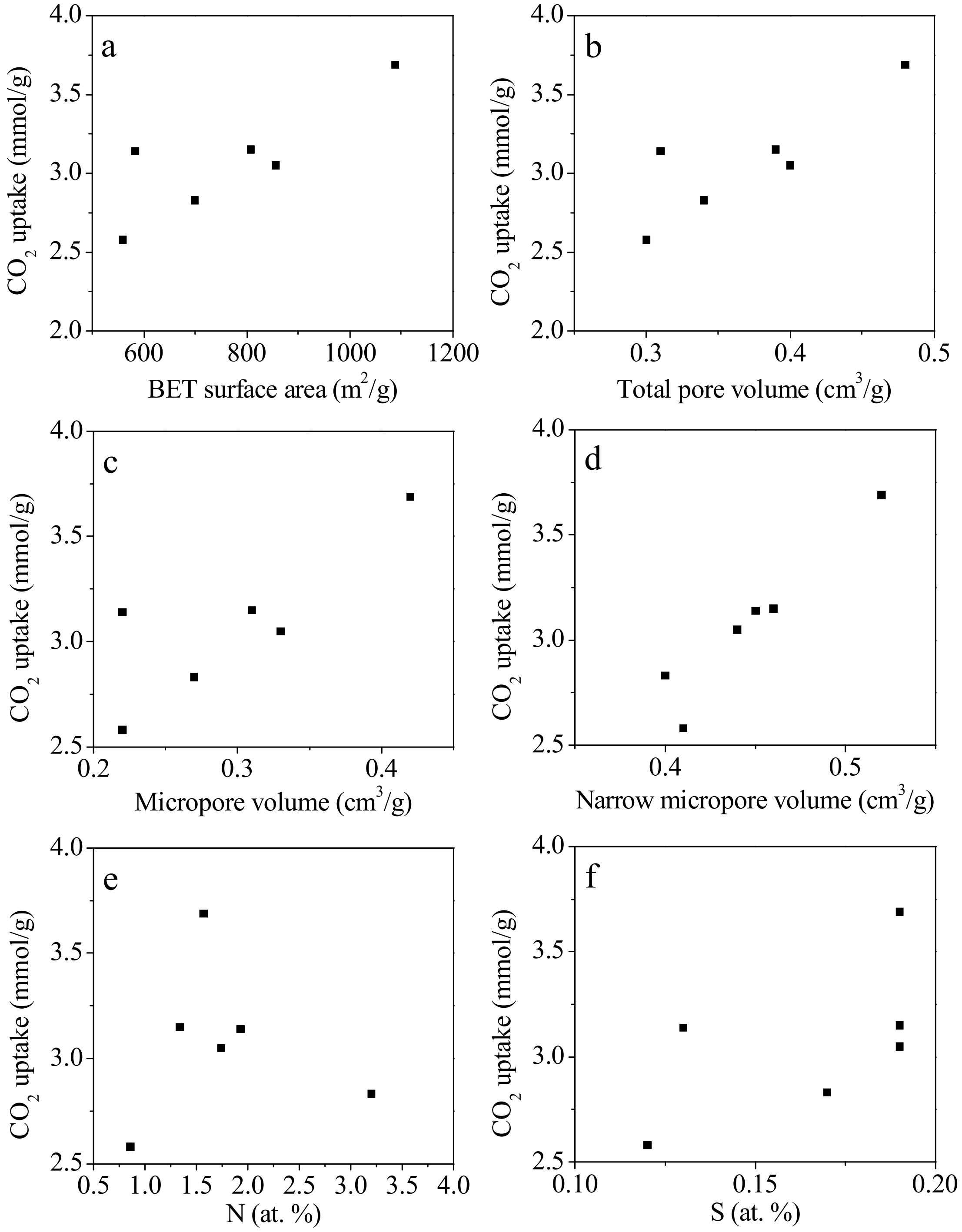

Figure S3. Plot of each porous properties characteristics (a) S_{BET}, (b) V_o, (c) V_t,(d) V_n , (e) nitrogen and (f) sulfur content versus CO_2 uptake.

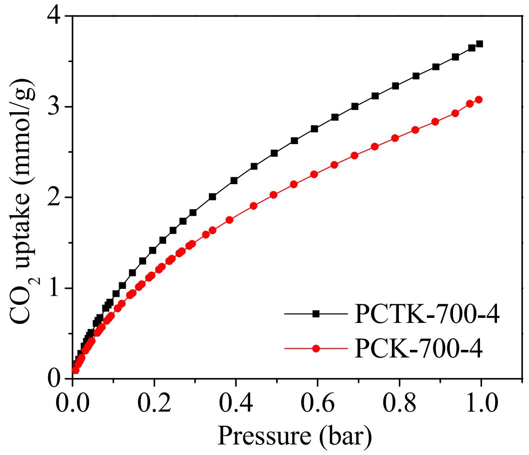

Figure S4. CO_2 adsorption isotherms of PCTK-700-4 vs PCK-700-4 at 25°C and 1 bar

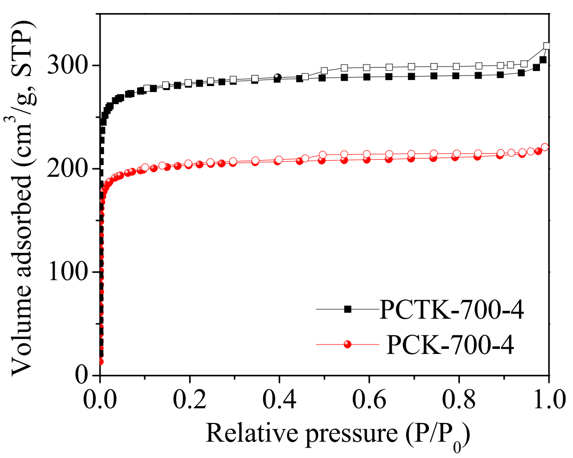

Figure S5. N_2 sorption isotherms of PCTK-700-4 vs PCK-700-4

Table S1. Comparison of the CO_2 adsorption (25 °C and 1 bar) for different sorbents

Sample	CO_2 uptake (mmol/g)	Ref.
AA750	2.7	S1
GEPM-1	2.5	S2
GMNO-4	2.6	S3
GTCF-3	2.7	S4
MRF-2	2.5	S5
OM-CNS	3.0	S6
SU-MC	1.0	S7
NDPC-2-800	0.8	S8
COP-122	0.4	S9
ZIF-78	2.7	S10
C-PP-750-1	2.6	S11
4-FSAC-HPC	3.3	S12
PSK-2-650	3.5	S13
Zeolite 13x	4.6	S14
Mg-MOF-74	7.1	S15
UiO-66(Zr)-$(OH)_2$	6.2	S16
PCTK-700-4	3.7	This study

References

S1. Balahmar, N.; Mitchell, A. C.; Mokaya, R., Generalized Mechanochemical Synthesis of Biomass-Derived Sustainable Carbons for High Performance CO_2 Storage. *Adv. Energy Mater.* **2015,** *5*, (22), 1500867.

S2. Sui, Z.Y.; Cui, Y.; Zhu, J.H.; Han, B.H., Preparation of Three-Dimensional Graphene Oxide–Polyethylenimine Porous Materials as Dye and Gas Adsorbents. *ACS Appl. Mater. Interfaces* **2013,** *5*, (18), 9172-9179.

S3. Zhou, D.; Liu, Q.; Cheng, Q.; Zhao, Y.; Cui, Y.; Wang, T.; Han, B., Graphene-manganese oxide hybrid porous material and its application in carbon dioxide adsorption. *Chin. Sci. Bull.* **2012,** *57*, (23), 3059-3064.

S4. Zhou, D.; Cheng, Q.Y.; Cui, Y.; Wang, T.; Li, X.; Han, B. H., Graphene-terpyridine

complex hybrid porous material for carbon dioxide adsorption. *Carbon* **2014,** *66*, 592-598.

S5. Zhou, H.; Xu, S.; Su, H.; Wang, M.; Qiao, W.; Ling, L.; Long, D., Facile preparation and ultra-microporous structure of melamine-resorcinol-formaldehyde polymeric microspheres. *Chem. Commun.* **2013,** *49*, (36), 3763-3765.

S6. Zheng, L.; Li, W. B.; Chen, J. L., Nitrogen doped hierarchical activated carbons derived from polyacrylonitrile fibers for CO2 adsorption and supercapacitor electrodes. *RSC Adv.* **2018,** *8*, (52), 29767-29774.

S7. To, J. W. F.; He, J.; Mei, J.; Haghpanah, R.; Chen, Z.; Kurosawa, T.; Chen, S.; Bae, W.-G.; Pan, L.; Tok, J. B. H.; Wilcox, J.; Bao, Z., Hierarchical N-Doped Carbon as CO_2 Adsorbent with High CO_2 Selectivity from Rationally Designed Polypyrrole Precursor. *J. Am. Chem. Soc.* **2016,** 138, (3), 1001-1009.

S8. Fu, N.; Wei, H.-M.; Lin, H.-L.; Li, L.; Ji, C.-H.; Yu, N.-B.; Chen, H.-J.; Han, S.; Xiao, G.-Y., Iron Nanoclusters as Template/Activator for the Synthesis of Nitrogen Doped Porous Carbon and Its CO_2 Adsorption Application. *ACS Appl. Mater. Interfaces* **2017,** 9, (11), 9955-9963.

S9. Dogan, N. A.; Ozdemir, E.; Yavuz, C. T., Direct Access to Primary Amines and Particle Morphology Control in Nanoporous CO_2 Sorbents. *ChemSusChem* **2017,** 10, (10), 2130-2134.

S10. Furukawa, H.; Yaghi, O. M., Storage of Hydrogen, Methane, and Carbon Dioxide in Highly Porous Covalent Organic Frameworks for Clean Energy Applications. *J. Am. Chem. Soc.* **2009,** *131*, (25), 8875-8883.

S11. Liu, C.; Zhi, Y.; Yu, Q.; Tian, L.; Demir, M.; Colak, S. G.; Farghaly, A. A.; Wang, L.; Hu, X., Sulfur-Enriched Nanoporous Carbon: A Novel Approach to CO_2 Adsorption. *ACS Appl. Nano Mater.* **2024,** 7, (5), 5434-5441.

S12. Miao, Z.; Wu, J.; Qiu, G.; Guo, Z.; Zhao, X.; Zhang, Y., Solving two industrial waste issues simultaneously: Coal gasification fine slag-based hierarchical porous composite with enhanced CO_2 adsorption performance, *Sci. Total Environ.* **2022**, 821, 153347.

S13. Zhang, Y.; Wei, Z.; Liu, X.; Liu, F.; Yan, Z.; Zhou, S.; Wang, J.; Deng, S., Synthesis of palm sheath derived-porous carbon for selective CO_2 adsorption, *RSC Adv.* **2022**, 12, 8592-8599.

S14. Cavenati, S.; Grande, C. A.; Rodrigues, A. E., Adsorption Equilibrium of Methane, Carbon Dioxide, and Nitrogen on Zeolite 13X at High Pressures. *J. Chem. Eng. Data* **2004,** 49, (4), 1095-1101.

S15. An, H.; Tian, W.; Lu, X.; Yuan, H.; Yang, L.; Zhang, H.; Shen, H.; Bai, H., Boosting the CO_2 adsorption performance by defect-rich hierarchical porous Mg-MOF-74. *Chem. Eng. J.* **2023,** 469, 144052.

S16. Vo, T. K.; Nguyen, V. C.; Quang, D. T.; Park, B. J.; Kim, J., Formation of structural defects within UiO-66(Zr)-$(OH)_2$ framework for enhanced CO_2 adsorption using a microwave-assisted continuous-flow tubular reactor. *Microporous Mesoporous Mater.* **2021,** 312, 110746.
